# Treatment-emergent adverse events occurring early in the treatment course of cladribine tablets in two phase 3 trials in multiple sclerosis

**DOI:** 10.1177/20552173211024298

**Published:** 2021-07-13

**Authors:** Jiwon Oh, Bryan Walker, Gavin Giovannoni, Dominic Jack, Fernando Dangond, Axel Nolting, Julie Aldridge, Lori A Lebson, Thomas P Leist

**Affiliations:** Division of Neurology, Department of Medicine, St Michael’s Hospital, University of Toronto, Toronto, ON, Canada; Duke University School of Medicine, Durham, NC, USA; Blizard Institute, Barts and The London School of Medicine and Dentistry, Queen Mary University of London, London, UK; the healthcare business of Merck KGaA, Darmstadt, Germany; EMD Serono, Billerica, MA, USA; the healthcare business of Merck KGaA, Darmstadt, Germany; EMD Serono, Billerica, MA, USA; EMD Serono, Rockland, MA, USA; Comprehensive Multiple Sclerosis Center, Jefferson University, Philadelphia, PA, USA

**Keywords:** Multiple sclerosis, disease modifying therapies, cladribine tablets, treatment-emergent adverse events, CLARITY, ORACLE-MS

## Abstract

**Background:**

Treatment-emergent adverse events (TEAEs) that occur close to treatment initiation may negatively affect overall tolerability and adherence. It is important to develop a clear understanding of potential early TEAEs after initiating treatment with cladribine tablets.

**Objective:**

To identify TEAEs that begin early in the course of treatment in patients enrolled in CLARITY and ORACLE-MS studies.

**Methods:**

This *post hoc* analysis of CLARITY and ORACLE-MS safety populations assessed the incidence of TEAEs, serious TEAEs, drug-related TEAEs, and TEAEs leading to discontinuation in patients receiving cladribine tablets or placebo within 2, 6, and 12 weeks after treatment initiation.

**Results:**

By Week 12, 61.3% of patients treated with cladribine tablets 3.5 mg/kg and 55.2% treated with placebo experienced a TEAE. More patients receiving cladribine tablets versus placebo experienced a drug-related TEAE by Week 12 (34.7% vs. 23.2%). The most common TEAEs reported with cladribine tablets were: headache (7.2%), lymphopenia (6.8%), and nausea (6.0%). Patients receiving cladribine tablets and placebo reported similar proportions of serious TEAEs (2.2% vs. 1.7%) and TEAEs leading to treatment discontinuation (1.6% vs. 1.4%).

**Conclusion:**

Cladribine tablets were well tolerated during the first 12 weeks as evidenced by a low incidence of TEAEs leading to treatment discontinuation.

## Introduction

Treatment adherence is a primary determinant of disease-modifying therapy (DMT) efficacy, but remains a key challenge among people with multiple sclerosis (PwMS).^1,2^ Real-world studies have shown that 25–40% of PwMS prescribed an injectable DMT and ∼30% prescribed an oral DMT discontinue therapy within five years after initiation.^3^ In MS, adherence to DMTs has been associated with a decreased rate of relapses and MS-related emergency room visits, slower disease progression, and an improvement in quality of life.^2,4^ Many factors influence adherence to DMTs in MS, including cognitive functioning, route of drug administration, convenience of use, provider/caregiver support, and efficacy.^5^ Tolerability and adherence to DMTs are also influenced by treatment-emergent adverse events (TEAEs) which may begin soon after initiation.^5^

Cladribine tablets 10 mg (3.5 mg/kg cumulative dose over 2 years; referred to as cladribine tablets 3.5 mg/kg), represent the first short-course oral DMT approved in more than 80 countries for various indications related to relapsing forms of MS (RMS).^6,7^ For the approved 2-year dose regimen, patients receive a cumulative dose of cladribine tablets 3.5 mg/kg at 1.75 mg/kg per treatment year given over 2 weeks in each year (4–5 consecutive treatment days each week).^6,7^

The safety and efficacy of cladribine tablets have been demonstrated in the pivotal CLARITY (NCT00213135) and ORACLE-MS (NCT00725985) studies in patients with relapsing-remitting MS (RRMS) and a first clinical demyelinating event (FCDE) at high risk of converting to MS, respectively.^8,9^ At the 3.5 mg/kg dosage, the most frequent TEAEs reported in ≥10% of patients receiving cladribine tablets during the two-year trial duration of both CLARITY and ORACLE-MS were headache, nasopharyngitis, lymphopenia, nausea, and upper respiratory tract infection. In addition, the overall frequency of treatment discontinuation due to TEAEs was low with cladribine tablets 3.5 mg/kg and placebo (CLARITY: 3.5% vs. 2.1%; ORACLE-MS: 5% vs. 2%, respectively).^8,9^ However, it is unclear whether the TEAEs occurred early in treatment since the TEAEs reported to date have been cumulative overall rates.

This *post hoc* analysis sought to identify TEAEs that occurred within 12 weeks after the commencement of treatment in patients enrolled in the Phase 3 CLARITY and ORACLE-MS clinical trials.

## Materials and methods

### Trial design

CLARITY and ORACLE-MS were Phase 3, double-blind, randomized, placebo-controlled, multicenter, 96-week studies of cladribine tablets in patients with RRMS and a FCDE, respectively. Study design details have been published previously.^8,9^ In brief, CLARITY included patients with a diagnosis of RRMS,^10^ lesions consistent with MS on magnetic resonance imaging (MRI),^11^ ≥1 relapse ≤12 months prior to enrollment, and an Expanded Disability Status Scale (EDSS) score of ≤5.5.^8^ ORACLE-MS included patients aged 18–55 years with a FCDE that occurred ≤75 days prior to screening, ≥2 clinically silent lesions of ≥3 mm on a T2-weighted brain MRI scan, and an EDSS score ≤5.^9^ Both studies were conducted in accordance with relevant clinical guidelines and were approved by independent ethics committees. All patients provided written informed consent.

In both CLARITY and ORACLE-MS, patients were randomly assigned (1:1:1 ratio) to receive cladribine tablets 3.5 mg/kg or 5.25 mg/kg (cumulative dosage over 2 years) or placebo.^8,9^ For the cladribine tablets 3.5 mg/kg dose regimen, 10 mg tablets were given over two weeks, administered at 0.875 mg/kg/week over 4–5 consecutive days starting on Day 1 of Weeks 1 and 5 of Year 1, followed by two treatment weeks at Weeks 48 and 52 of Year 2.

The primary and key secondary efficacy endpoints assessed in CLARITY were relapse rate at 96 weeks and proportion of patients who were relapse-free, time to sustained progression of disability, and gadolinium-enhancing T1 and new/enlarging T2 lesion activity on MRI scans, respectively.^8^ The primary and key secondary endpoints assessed in ORACLE-MS were time to conversion to MS based on the Poser criteria and 2005 McDonald criteria, respectively.^9^

### Post hoc analysis

The focus of this *post hoc* analysis of data from CLARITY and ORACLE-MS was to identify TEAEs associated with placebo and cladribine tablets 3.5 mg/kg during the first 12 weeks, which encompasses the full dosing period in the first treatment year. As cladribine is quickly eliminated from the body, with a terminal half-life of ∼1 day,^6^ any observed effects are the result of long-term pharmacodynamic changes. The analysis focused on patients receiving cladribine tablets 3.5 mg/kg and placebo, as 3.5 mg/kg is the recommended dosage for the treatment of RMS (where approved). The analysis set included the pooled CLARITY safety population (Weeks 0–96) and the ORACLE-MS safety population during the initial treatment period (study Day 1 to end of Week 96).

Study endpoints of interest in this analysis included the incidence of any TEAE, any serious TEAE, any TEAE leading to treatment discontinuation, and any drug-related TEAE (according to the judgement of the investigator) within 2, 6 and 12 weeks after the commencement of treatment. The severity classification (mild, moderate, or severe) of a TEAE was determined by the investigator which was not based on pre-defined criteria. A serious TEAE was defined as any adverse event (AE) that occurred at any treatment dose that resulted in death; was life-threatening; required hospitalization or prolongation of existing hospitalization; resulted in persistent or significant disability or incapacity; was a congenital anomaly or birth defect; or was considered medically important (i.e., a serious adverse drug experience that might jeopardize the patient and might require medical or surgical intervention to prevent one of the aforementioned outcomes). Endpoints were summarized by severity, MedDRA Primary System Organ Class (SOC) and Preferred Term (PT), and all AEs were coded according to the MedDRA dictionary Version 11.0 as per CLARITY and ORACLE-MS study protocols.

The TEAE evaluation time points at Weeks 2 and 6 correspond to the completion of active treatment in Weeks 1 and 5, respectively, and reflect the completion of treatment course for the approved cladribine tablets 3.5 mg/kg dosage for Year 1. TEAE evaluation time points show the cumulative TEAEs at each time point. For each event analyzed, a patient was counted once by TEAE for the overview table, and once by severity (taking the worst severity for the respective TEAE) for the summary tables. All analyses were performed using SAS® software version 9.4 or higher. Formal statistical testing to compare the cladribine tablets 3.5 mg/kg and placebo treatment arms was not performed because this *post hoc* analysis was not designed to address multiplicity, which increases the probability of mistakenly declaring a difference.^12^ In lieu of performing formal statistical testing, clinically meaningful differences in TEAE frequency between treatment arms were identified, which was defined as a ≥5% absolute change.

## Results

### Patient disposition

In the CLARITY/ORACLE-MS combined analysis, 636 patients received cladribine tablets 3.5 mg/kg and 641 patients received placebo. More than 85% of patients across treatment groups remained on study for at least 12 weeks in CLARITY (cladribine tablets 3.5 mg/kg: 425 [98.8%]; placebo: 428 [98.4%]) and ORACLE-MS (cladribine tablets 3.5 mg/kg: 191 [92.7%]; placebo: 177 [85.9%]; Table 1). The lower percentage of ORACLE-MS patients in the placebo group remaining in the study was due to a higher proportion of patients who converted to MS versus those who received active treatment and transitioned to the open-label maintenance period part of the study.^9^

**Table 1.** Patient disposition.

	CLARITY		ORACLE-MS	
	Placebo (N = 435)	Cladribine tablets 3.5 mg/kg (N = 430)	Placebo (N = 206)	Cladribine tablets 3.5 mg/kg (N = 206)
Patients who discontinued study medication, n (%)	58 (13.3)	35 (8.1)	31 (15.0)	48 (23.3)

Time on study (weeks)				
At least 2 weeks, n (%)	434 (99.8)	428 (99.5)	195 (94.7)	198 (96.1)
At least 6 weeks, n (%)	431 (99.1)	426 (99.1)	189 (91.7)	195 (94.7)
At least 12 weeks, n (%)	428 (98.4)	425 (98.8)	177 (85.9)	191 (92.7)

### TEAEs

In combined analyses, a similar proportion of patients experienced a TEAE across treatment groups (cladribine tablets 3.5 mg/kg: 81%; placebo: 75%; Table 2). Within the first 2 weeks, approximately one-third of patients in each treatment group experienced a TEAE (cladribine tablets 3.5 mg/kg: 32.4%; placebo: 30.6%), which increased to >50% by Week 12 (cladribine tablets 3.5 mg/kg: 61.3%; placebo: 55.2%). The absolute increase of 6.1% in the active treatment group over the placebo group by Week 12 was considered clinically meaningful.

**Table 2.** Overall incidence of adverse events – Combined CLARITY and ORACLE-MS.

	Placebo (N = 641)		Cladribine tablets 3.5 mg/kg (N = 636)	
n (%)	Patients	Events	Patients	Events
Any TEAE	481 (75.0)	3216 (100.0)	515 (81.0)	3867 (100.0)
Occurring in the first 2 weeks	196 (30.6)	353 (11.0)	206 (32.4)	375 (9.7)
Occurring in the first 6 weeks	282 (44.0)	716 (22.3)	305 (48.0)	769 (19.9)
Occurring in the first 12 weeks*	354 (55.2)	1144 (35.6)	390 (61.3)	1271 (32.9)
Any drug-related TEAE	226 (35.3)	732 (100.0)	335 (52.7)	1251 (100.0)
Occurring in the first 2 weeks	78 (12.2)	130 (17.8)	100 (15.7)	183 (14.6)
Occurring in the first 6 weeks*	109 (17.0)	229 (31.3)	169 (26.6)	361 (28.9)
Occurring in the first 12 weeks*	149 (23.2)	352 (48.1)	221 (34.7)	533 (42.6)
Any serious TEAE	50 (7.8)	78 (100.0)	59 (9.3)	89 (100.0)
Occurring in the first 2 weeks	2 (0.3)	2 (2.6)	2 (0.3)	3 (3.4)
Occurring in the first 6 weeks	7 (1.1)	8 (10.3)	7 (1.1)	9 (10.1)
Occurring in the first 12 weeks	11 (1.7)	14 (17.9)	14 (2.2)	19 (21.3)
Any TEAE leading to treatment discontinuation	15 (2.3)	21 (100.0)	25 (3.9)	26 (100.0)
Occurring in the first 2 weeks	3 (0.5)	3 (14.3)	2 (0.3)	2 (7.7)
Occurring in the first 6 weeks	6 (0.9)	7 (33.3)	6 (0.9)	6 (23.1)
Occurring in the first 12 weeks	9 (1.4)	11 (52.4)	10 (1.6)	11 (42.3)

TEAE: treatment-emergent adverse event.

*Clinically meaningful difference (≥5% absolute difference) between placebo and cladribine tablets 3.5 mg/kg.

Among TEAEs experienced by patients in the first 12 weeks of treatment with cladribine tablets 3.5 mg/kg or placebo, most were mild in severity. During the first 2 weeks (68.0% vs. 68.4%), 6 weeks (58.4% vs. 61.0%), and 12 weeks (54.4% vs. 53.7%) of patients reported mild TEAEs with cladribine tablets 3.5 mg/kg versus placebo, respectively; however, the proportion of patients with a mild TEAE decreased at a similar rate over time between the treatment groups. The most commonly reported TEAEs during the first 12 weeks after initiating cladribine tablets 3.5 mg/kg or placebo, respectively, were headache (18.4% vs. 15.1%), nausea (8.0% vs. 4.5%), lymphopenia (6.8% vs. 0.5%), and nasopharyngitis (5.5% vs. 6.4%; Table 3). During the 12-week period, 3.0% of patients in the cladribine tablets 3.5 mg/kg group and 1.7% of patients in the placebo group experienced a TEAE of severe intensity; of the commonly reported TEAEs (Table 3), this was noted for headache (0.8% vs. 0.3%), nausea (0.2% vs. 0%), diarrhea (0.2% vs. 0%), fatigue (0.2% vs. 0%), and arthralgia (0.2% vs. 0%) for cladribine tablets 3.5 mg/kg versus placebo groups, respectively.

**Table 3.** Most common TEAEs (reported in ≥2% of patients in the cladribine tablets 3.5 mg/kg group; ordered by most common in cladribine tablets 3.5 mg/kg group) – Combined CLARITY and ORACLE-MS.

n (%)	Placebo (N = 641)	Cladribine tablets 3.5 mg/kg (N = 636)
First 2 weeks		
Patients with any TEAE	196 (30.6)	206 (32.4)
Headache	53 (8.3)	57 (9.0)
Nausea	21 (3.3)	31 (4.9)
First 6 weeks		
Patients with any TEAE	282 (44.0)	305 (48.0)
Headache	76 (11.9)	94 (14.8)
Nausea	24 (3.7)	41 (6.4)
Nasopharyngitis	22 (3.4)	21 (3.3)
Diarrhea	19 (3.0)	19 (3.0)
Influenza-like illness	7 (1.1)	18 (2.8)
Fatigue	20 (3.1)	16 (2.5)
Lymphopenia	–	16 (2.5)
Upper respiratory tract infection	15 (2.3)	15 (2.4)
First 12 weeks		
Patients with any TEAE*	354 (55.2)	390 (61.3)
Headache	97 (15.1)	117 (18.4)
Nausea	29 (4.5)	51 (8.0)
Lymphopenia*	3 (0.5)	43 (6.8)
Nasopharyngitis	41 (6.4)	35 (5.5)
Upper respiratory tract infection	27 (4.2)	29 (4.6)
Diarrhea	24 (3.7)	26 (4.1)
Abdominal pain (upper)	11 (1.7)	21 (3.3)
Influenza-like illness	14 (2.2)	21 (3.3)
Fatigue	28 (4.4)	20 (3.1)
Arthralgia	16 (2.5)	17 (2.7)
Dizziness	13 (2.0)	16 (2.5)
Pharyngolaryngeal pain	17 (2.7)	15 (2.4)
Abdominal pain	10 (1.6)	13 (2.0)
Alopecia	5 (0.8)	13 (2.0)

TEAEs: treatment-emergent adverse events.

Patients with >1 occurrence of the same adverse event are counted at the maximal intensity to the study treatment reported for that patient.

*Clinically meaningful difference (≥5% absolute difference) between placebo and cladribine tablets 3.5 mg/kg.

Herpetic infections (herpes simplex, oral herpes, genital herpes, and herpes virus infection) were reported by Week 12 in patients treated with cladribine tablets 3.5 mg/kg and in those who received placebo (Supplementary Table S1). By Week 12, herpes simplex was reported among 0.2% of patients treated either with cladribine tablets 3.5 mg/kg or placebo.

### Serious TEAEs

Few patients in either treatment group experienced serious TEAEs during the first 12 weeks (cladribine tablets 3.5 mg/kg: 2.2%; placebo: 1.7%), and the majority of events were reported in <1% of patients and were unique to each treatment group (Supplementary Table S2). The most commonly reported serious TEAEs with cladribine tablets 3.5 kg/mg were increased blood amylase, lipase and blood creatine phosphokinase, and injury from a fall (each occurring in 0.3% of patients). In placebo-treated patients, the most commonly reported serious TEAE was appendicitis, which occurred in 0.3% of patients.

### Drug-related TEAEs

A greater proportion of patients receiving cladribine tablets 3.5 mg/kg experienced drug-related TEAEs versus the placebo group during the first 2 weeks (15.7% vs. 12.2%), 6 weeks (26.6% vs. 17.0%), and 12 weeks (34.7% vs. 23.2%), respectively (Supplementary Table S3). The absolute frequency increases in drug-related TEAEs in the cladribine tablets group over the placebo group during the first 6 and 12 weeks were deemed clinically meaningful. The most commonly reported drug-related TEAEs by Week 12 with cladribine tablets 3.5 mg/kg were headache (7.2%), lymphopenia (6.8%), nausea (6.0%), and diarrhea (2.5%), and with placebo were headache (5.0%), nausea (2.8%), fatigue (2.3%), and diarrhea (2.0%). Most drug-related TEAEs by Week 12 were mild in severity (cladribine tablets 3.5 mg/kg: 54.8%; placebo: 59.1%). During this period, drug-related severe TEAEs were reported in 1.3% of patients in the cladribine tablets 3.5 mg/kg group and 0.6% in the placebo group with the most common being headache (0.5% vs. 0.2%, respectively). While the frequencies of the most common drug-related TEAEs were generally higher in patients treated with cladribine tablets 3.5 mg/kg versus placebo at each time point evaluated, the observed frequency of drug-related fatigue and diarrhea were similar in cladribine tablets versus placebo by Week 12 (fatigue: 1.9% vs. 2.3%; diarrhea: 2.5% vs. 2.0%).

Lymphopenia as a drug-related TEAE occurred more frequently with cladribine tablets 3.5 mg/kg (0.3%, 2.5%, and 6.8% of patients at Weeks 2, 6, and 12, respectively) versus placebo (0%, 0%, and 0.5% of patients at Weeks 2, 6, and 12, respectively), with the increase deemed clinically meaningful by Week 12 (Supplementary Table S3). All cases of lymphopenia were mild to moderate in severity during the first 12 weeks. By Week 12, drug-related leukopenia was reported in eight (1.3%) patients receiving cladribine tablets 3.5 mg/kg and no patients receiving placebo. Drug-related infections were low overall but reported by a slightly higher proportion of patients treated with cladribine tablets 3.5 mg/kg versus placebo during the first 12 weeks (influenza-like illness: 1.9% vs. 0.8%; nasopharyngitis: 1.7% vs. 0.6%; upper respiratory tract infection: 1.3% vs. 0.8%).

### TEAEs leading to treatment discontinuation

The proportion of patients experiencing TEAEs leading to discontinuation was low during the first 12 weeks and similar between groups (cladribine tablets 3.5 mg/kg: 1.6% of patients; placebo: 1.4% of patients) with each individual TEAE observed in <1% of patients (Supplementary Table S4). Of these patients, four (0.6%) from each treatment group discontinued during treatment (Weeks 1 and 5); in the cladribine tablets group, two (0.3%) discontinued in the period between the two treatments (Weeks 2–4); four (0.6%) from each treatment group discontinued after treatment completion (Weeks 6–12; Supplementary Table S5). Lymphopenia (moderate in severity) was the most common TEAE leading to treatment discontinuation with cladribine tablets 3.5 mg/kg and was reported in three (0.5%) patients. The first patient had lymphopenia that did not resolve after two months; lymphopenia coincided with a mild upper respiratory tract infection that lasted a month. The second patient was experiencing lymphopenia on the study start date, and discontinued the study on the same day. The third patient experienced mild headache at the start of the treatment; 28 days later, lymphopenia was reported, which resolved in six days. The only TEAEs leading to treatment discontinuation that were observed in both treatment groups during the first 12 weeks were increased alanine aminotransferase (ALT), reported in two (0.3%) patients receiving cladribine tablets 3.5 mg/kg and one (0.2%) receiving placebo, and aspartate aminotransferase reported in one (0.2%) patient in either treatment group.

## Discussion

**Figure 1.**
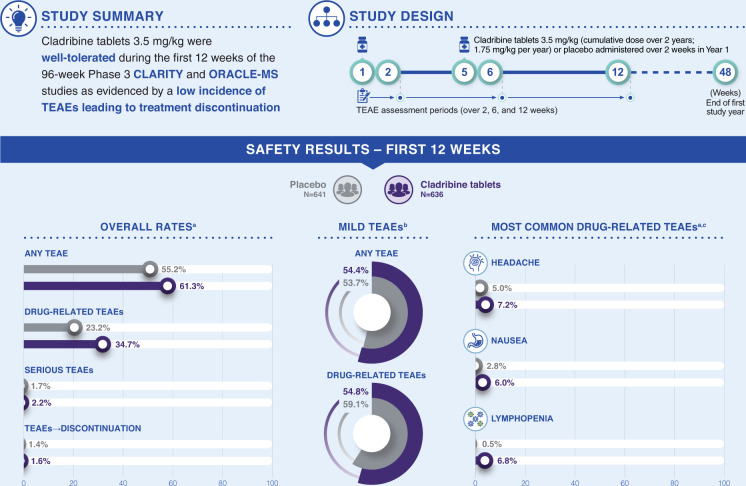
Study summary. TEAE: treatment-emergent adverse event. ^a^Proportion of patients relative to the overall populations in the placebo and cladribine tablets groups. ^b^Proportion relative to those with any TEAE or drug-related TEAEs. ^c^In >5% of patients in either treatment group.

The efficacy and safety of cladribine tablets 3.5 mg/kg have been demonstrated in the pivotal CLARITY and ORACLE-MS studies in patients with RRMS and a FCDE, respectively.^8,9^ Cladribine tablets 3.5 mg/kg is unique among available DMTs for relapsing forms of MS as it is a short-course, orally administered therapy that has demonstrated durable benefit without the need for chronic administration.^8,13^ While the short-course oral administration of cladribine tablets may be expected to facilitate treatment compliance among patients, tolerability is a key determinant of adherence to treatment regimens and this has not yet been fully investigated in cladribine tablets. This *post hoc* analysis of CLARITY and ORACLE-MS demonstrated a low incidence of TEAEs and low rate of TEAEs leading to treatment discontinuation shortly after commencement of cladribine tablets 3.5 mg/kg, suggesting that tolerability was not a significant issue in the pivotal clinical trials (Figure 1). The most frequent drug-related TEAEs of any severity in patients receiving cladribine tablets 3.5 mg/kg were headache, nausea, and lymphopenia during the first 12 weeks. The increase in the incidence of lymphopenia was greater and clinically meaningful in the cladribine tablets-treated versus placebo-treated groups (6.8% vs. 0.5%), which is consistent with its mechanism of action; however, the increase in frequency of nausea (6.0% vs. 2.8%) and headache (7.2% vs. 5.0%) was more moderate. The drug-related TEAE profile remains consistent with the overall established AE profile for cladribine tablets 3.5 mg/kg.^6^ A similarly low proportion of patients receiving cladribine tablets 3.5 mg/kg or placebo experienced a TEAE leading to treatment discontinuation and a serious TEAE during the first 12 weeks, with serious events unique to each treatment group.

As anticipated from the lymphocyte-reducing mechanism of action of cladribine, drug-related lymphopenia occurred more frequently with cladribine tablets 3.5 mg/kg than placebo during the first 12 weeks (6.8% vs. 0.5%, respectively); all cases were mild to moderate in severity and led to treatment discontinuation in 0.5% of patients receiving cladribine tablets 3.5 mg/kg. As cladribine tablets interrupt the central immune cascade involved in MS by a mechanism that involves a transient reduction in peripheral lymphocyte levels, patients are at an increased risk of lymphopenia and may be more susceptible to infection.^14^ By Week 12, drug-related infection rates (influenza-like illness, nasopharyngitis, and upper respiratory tract infection) were low (<2%) in both treatment groups (cladribine tablets 3.5 mg/kg vs. placebo: 1.3–1.9% vs. 0.6–0.8%) and no infections led to treatment discontinuation in patients receiving cladribine tablets 3.5 mg/kg.

Maintaining adherence to DMTs is typically challenging among PwMS because of TEAEs, particularly for DMTs that require chronic administration. Cladribine tablets have the benefit of short courses of therapy which may improve adherence; however, despite the short treatment course, adherence can be a concern if TEAEs that begin shortly after treatment initiation are frequent. The present analysis demonstrates that cladribine tablets 3.5 mg/kg were generally well tolerated among patients. While a greater proportion of patients receiving cladribine tablets 3.5 mg/kg versus placebo experienced any TEAE (61.3% vs. 55.2%, respectively) and drug-related TEAE (34.7% vs. 23.2%, respectively) by Week 12, a similar and low proportion of patients in each group discontinued treatment due to a TEAE (1.6% vs. 1.4%, respectively). The low rates of treatment discontinuation were a reflection of the observation that in cladribine tablets- or placebo-treated patients who experienced a TEAE, most reported TEAEs mild in severity (54.4% vs. 53.7%, respectively) and few reported TEAEs of severe intensity (3.0% vs. 1.7%). As such, the AEs that occurred were not sufficiently intolerable for most patients to consider treatment discontinuation. These results suggest that there were few drug tolerability issues with cladribine tablets 3.5 mg/kg during the first 12 weeks following completion of Year 1 dosing, and may facilitate adherence among PwMS.

While adherence to DMTs can also be influenced by the route and frequency of administration, real-world data suggest that adherence to oral maintenance DMTs (i.e., dimethyl fumarate, fingolimod, and teriflunomide) is similar to that of self-injectable DMTs.^3,15,16^ One key advantage of cladribine tablets 3.5 mg/kg treatment is the short-course oral administration regimen that results in durable clinical benefit for many patients, as demonstrated in the CLARITY and CLARITY Extension studies.^8,13^ In clinical practice, patients receive the complete yearly dose of cladribine tablets over two treatment weeks making them less likely to discontinue or be non-adherent to therapy compared with DMTs that are administered in constant regular intervals. The convenient dosing of cladribine tablets together with the finding that few patients experience tolerability issues during the short treatment regimen suggest that cladribine tablets may be relatively easily adhered to by PwMS compared with other DMTs.

This study has a number of limitations. First, this was a *post hoc* analysis of clinical trial data and does not fully evaluate tolerability of cladribine tablets as data were collected only at predetermined time points. Nonetheless, since information on AEs were collected at frequent time points after cladribine tablet administration, many tolerability issues were likely captured. Second, the impact of changes in lymphocyte levels over time on patient adherence to treatment was not fully evaluated. Previous studies have reported that cladribine tablets reduced lymphocytes soon after treatment initiation and the nadir of absolute lymphocyte count occurred at Week 9 in the first treatment year; this was followed by a steady increase in ALC that occurred over the next few months.^8,17^ While this 12-week study encompasses the ALC nadir period and reports treatment discontinuation due to lymphopenia (0.5%), the effect of gradual ALC increase beyond Week 12 on treatment adherence was not captured. However, as ALC recovered quickly to normal range after reaching nadir in the first treatment year,^8,17^ it is unlikely that a prolonged lymphocyte recovery time would be a factor preventing patients from continuing treatment in the second year. Finally, several other factors outside of AEs may impact patient adherence to treatment and therapy discontinuation.^5^ As such, although these results are informative, there is a need for real-world follow-up studies in patients with RMS receiving treatment with cladribine tablets 3.5 mg/kg, in order to evaluate all factors that influence adherence early in the treatment course.

## Conclusion

This *post hoc* analysis of data from two Phase 3 studies demonstrate that cladribine tablets 3.5 mg/kg were well tolerated during the first 12 weeks after treatment commencement (and after completion of the Year 1 dose) as evidenced by a low incidence of TEAEs and a low rate of AE-related treatment discontinuation. Overall, these data support the use of cladribine tablets as an effective treatment for eligible patients with relapsing MS, as it is a well-tolerated therapy that has the potential for durable benefit without the need for chronic administration, which can facilitate treatment adherence and enable optimization of clinical outcomes.

## Supplemental Material

sj-pdf-1-mso-10.1177_20552173211024298 - Supplemental material for Treatment-emergent adverse events occurring early in the treatment course of cladribine tablets in two phase 3 trials in multiple sclerosis Click here for additional data file. Supplemental material, sj-pdf-1-mso-10.1177_20552173211024298 for Treatment-emergent adverse events occurring early in the treatment course of cladribine tablets in two phase 3 trials in multiple sclerosis by Jiwon Oh, Bryan Walker, Gavin Giovannoni, Dominic Jack, Fernando Dangond, Axel Nolting, Julie Aldridge, Lori A Lebson and Thomas P Leist in Multiple Sclerosis Journal—Experimental, Translational and Clinical

## References

[1] Haase R Kullmann JS Ziemssen T. Therapy satisfaction and adherence in patients with relapsing-remitting multiple sclerosis: the THEPA-MS survey. Ther Adv Neurol Disord 2016; 9: 250–263. 27366231 10.1177/1756285616634247 PMC4916516

[2] Tan H Cai Q Agarwal S , et al. Impact of adherence to disease-modifying therapies on clinical and economic outcomes among patients with multiple sclerosis. Adv Ther 2011; 28: 51–61. 21153000 10.1007/s12325-010-0093-7

[3] Burks J Marshall TS Ye X. Adherence to disease-modifying therapies and its impact on relapse, health resource utilization, and costs among patients with multiple sclerosis. Clinicoecon Outcomes Res 2017; 9: 251–260. 28496344 10.2147/CEOR.S130334 PMC5417677

[4] Munsell M Frean M Menzin J , et al. An evaluation of adherence in patients with multiple sclerosis newly initiating treatment with a self-injectable or an oral disease-modifying drug. Patient Prefer Adherence 2017; 11: 55–62. 28115831 10.2147/PPA.S118107 PMC5221550

[5] Higuera L Carlin CS Anderson S. Adherence to disease-modifying therapies for multiple sclerosis. J Manag Care Spec Pharm 2016; 22: 1394–1401. 27882830 10.18553/jmcp.2016.22.12.1394 PMC10397889

[6] *Mavenclad [package insert]*. Rockland, MA: EMD Serono, Inc.; 2019.

[7] Mavenclad (summary of product characteristics). Amsterdam, The Netherlands: Merck Europe B.V., 2020.

[8] Giovannoni G Comi G Cook S , et al. A placebo-controlled trial of oral cladribine for relapsing multiple sclerosis. N Engl J Med 2010; 362: 416–426. 20089960 10.1056/NEJMoa0902533

[9] Leist TP Comi G Cree BA , et al. Effect of oral cladribine on time to conversion to clinically definite multiple sclerosis in patients with a first demyelinating event (ORACLE MS): a phase 3 randomised trial. Lancet Neurol 2014; 13: 257–267. 24502830 10.1016/S1474-4422(14)70005-5

[10] McDonald WI Compston A Edan G , et al. Recommended diagnostic criteria for multiple sclerosis: guidelines from the international panel on the diagnosis of multiple sclerosis. Ann Neurol 2001; 50: 121–127. 11456302 10.1002/ana.1032

[11] Fazekas F Barkhof F Filippi M , et al. The contribution of magnetic resonance imaging to the diagnosis of multiple sclerosis. Neurology 1999; 53: 448–456. 10449103 10.1212/wnl.53.3.448

[12] Harrington D D’Agostino RB Gatsonis C , et al. New guidelines for statistical reporting in the journal. N Engl J Med 2019; 381: 285–286. 31314974 10.1056/NEJMe1906559

[13] Giovannoni G Soelberg Sorensen P Cook S , et al. Safety and efficacy of cladribine tablets in patients with relapsing-remitting multiple sclerosis: results from the randomized extension trial of the CLARITY study. Mult Scler 2018; 24: 1594–1604. 28870107 10.1177/1352458517727603

[14] Deeks ED. Cladribine tablets: a review in relapsing MS. CNS Drugs 2018; 32: 785–796. 30105527 10.1007/s40263-018-0562-0 PMC6353806

[15] Lanzillo R Prosperini L Gasperini C , et al. A multicentRE observational analysiS of PErsistenCe to Treatment in the new multiple sclerosis era: the RESPECT study. J Neurol 2018; 265: 1174–1183. 29549468 10.1007/s00415-018-8831-x

[16] Ferraro D Camera V Baldi E , et al. First-line disease-modifying drugs in relapsing-remitting multiple sclerosis: an Italian real-life multicenter study on persistence. Curr Med Res Opin 2018; 34: 1803–1807. 29526118 10.1080/03007995.2018.1451311

[17] Comi G Cook S Giovannoni G , et al. Effect of cladribine tablets on lymphocyte reduction and repopulation dynamics in patients with relapsing multiple sclerosis. Mult Scler Relat Disord 2019; 29: 168–174. 30885375 10.1016/j.msard.2019.01.038

