# Supplementary material for: Treatment-emergent adverse events occurring early in the treatment course of cladribine tablets in two phase 3 trials in multiple sclerosis
Source: Mult Scler J Exp Transl Clin. 2021 Jul 13;7(3):20552173211024298. doi: 10.1177/20552173211024298 (PMC8283088; doi:10.1177/20552173211024298)
Supplement: sj-pdf-1-mso-10.1177_20552173211024298 - Supplemental material for Treatment-emergent adverse events occurring early in the treatment course of cladribine tablets in two phase 3 trials in multiple sclerosis [file sj-pdf-1-mso-10.1177_20552173211024298.pdf]

Treatment-emergent adverse events occurring early in the treatment course of cladribine tablets in two Phase 3 trials in multiple sclerosis

SUPPLEMENTARY TABLES

Supplemental Table S1. Incidence of herpetic infections – Combined CLARITY and ORACLE-MS

n (%)	Placebo (N=641)			Cladribine tablets 3.5 mg/kg (N=636)		
	**First 2 weeks**	**First 6 weeks**	**First 12 weeks**	**First 2 weeks**	**First 6 weeks**	**First 12 weeks**
Oral herpes	2 (0.3)	2 (0.3)	5 (0.8)	1 (0.2)	2 (0.3)	7 (1.1)
Herpes zoster	-	-	-	-	-	-
Herpes simplex	-	1 (0.2)	1 (0.2)	1 (0.2)	1 (0.2)	1 (0.2)
Genital herpes	-	-	-	-	-	1 (0.2)
Herpes virus infection	-	-	-	-	-	1 (0.2)

Supplementary Table S2. Any serious TEAEs in the first 2, 6, and 12 weeks – Combined CLARITY and ORACLE-MS (ordered by system organ class and preferred term)

n (%)	Placebo (N=641)			Cladribine tablets 3.5 mg/kg (N=636)		
	First 2 weeks	First 6 weeks	First 12 weeks	First 2 weeks	First 6 weeks	First 12 weeks
Patients with any serious TEAE	2 (0.3)	7 (1.1)	11 (1.7)	2 (0.3)	7 (1.1)	14 (2.2)
Eye disorders	-	-	1 (0.2)	-	-	-
Eyelid ptosis	-	-	1 (0.2)	-	-	-
Gastrointestinal disorders	-	1 (0.2)	2 (0.3)	-	-	-
Mechanical ileus	-	1 (0.2)	1 (0.2)	-	-	-
Pancreatitis acute	-	-	1 (0.2)	-	-	-
Hepatobiliary disorders	-	-	-	-	1 (0.2)	1 (0.2)
Hepatitis toxic	-	-	-	-	1 (0.2)	1 (0.2)
Infections and infestations	1 (0.2)	3 (0.5)	3 (0.5)	-	1 (0.2)	2 (0.3)
Appendicitis	1 (0.2)	2 (0.3)	2 (0.3)	-	-	-
Myocarditis bacterial	-	1 (0.2)	1 (0.2)	-	-	-

Pilonidal cyst	-	-	-	-	1 (0.2)	1 (0.2)
Pneumonia	-	1 (0.2)	1 (0.2)	-	-	-
Pyelonephritis	-	-	-	-	-	1 (0.2)
Injury, poisoning, and procedural complications	-	-	1 (0.2)	-	-	4 (0.6)
Clavicle fracture	-	-	1 (0.2)	-	-	-
Fall	-	-	-	-	-	2 (0.3)
Femoral neck fracture	-	-	-	-	-	1 (0.2)
Overdose	-	-	-	-	-	1 (0.2)
Road traffic accident	-	-	1 (0.2)	-	-	-
Thoracic vertebral fracture	-	-	-	-	-	1 (0.2)
Upper limb fracture	-	-	-	-	-	1 (0.2)
Investigations	-	1 (0.2)	1 (0.2)	2 (0.3)	4 (0.6)	5 (0.8)
Alanine aminotransferase increased	-	-	-	-	1 (0.2)	1 (0.2)
Blood amylase increased	-	-	-	1 (0.2)	2 (0.3)	2 (0.3)
Blood creatine phosphokinase increased	-	-	-	1 (0.2)	1 (0.2)	2 (0.3)

Blood uric acid increased	-	1 (0.2)	1 (0.2)	-	-	-
Lipase increased	-	-	-	1 (0.2)	2 (0.3)	2 (0.3)
Musculoskeletal and connective tissue disorders	-	-	-	-	1 (0.2)	1 (0.2)
Arthropathy	-	-	-	-	1 (0.2)	1 (0.2)
Neoplasms (benign, malignant, and unspecified)	-	-	1 (0.2)	-	-	-
Thyroid neoplasm	-	-	1 (0.2)	-	-	-
Nervous system disorder	-	-	1 (0.2)	-	-	1 (0.2)
Altered state of consciousness	-	-	-	-	-	1 (0.2)
Facial spasm	-	-	1 (0.2)	-	-	-
Pregnancy, puerperium, and perinatal conditions	1 (0.2)	1 (0.2)	1 (0.2)	-	-	1 (0.2)
Abortion spontaneous	1 (0.2)	1 (0.2)	1 (0.2)	-	-	-
Ectopic pregnancy	-	-	-	-	-	1 (0.2)
Respiratory, thoracic, and mediastinal disorders	-	1 (0.2)	1 (0.2)	-	-	-

Tracheal mass	-	1 (0.2)	1 (0.2)	-	-	-

TEAE, treatment-emergent adverse event

Supplementary Table S3. Most common drug-related TEAEs in the first 2, 6, and 12 weeks – Combined CLARITY and ORACLE-MS (≥2% of patients in the cladribine tablets 3.5 mg/kg group; ordered by most common in cladribine tablets 3.5 mg/kg)

n (%)	Placebo (N=641)	Cladribine tablets 3.5 mg/kg (N=636)
**First 2 weeks**		
Patients with any drug-related TEAE	78 (12.2)	100 (15.7)
Nausea	14 (2.2)	26 (4.1)
Headache	16 (2.5)	25 (3.9)
**First 6 weeks**		
Patients with any drug-related TEAE*	109 (17.0)	169 (26.6)
Headache	22 (3.4)	39 (6.1)
Nausea	15 (2.3)	32 (5.0)
Lymphopenia	-	16 (2.5)
Diarrhea	10 (1.6)	15 (2.4)
**First 12 weeks**		
Patients with any drug-related TEAE*	149 (23.2)	221 (34.7)
Headache	32 (5.0)	46 (7.2)

Treatment-emergent adverse events occurring early in the treatment course of cladribine tablets in two Phase 3 trials in multiple sclerosis

Lymphopenia*	3 (0.5)	43 (6.8)
Nausea	18 (2.8)	38 (6.0)
Diarrhea	13 (2.0)	16 (2.5)
Abdominal pain (upper)	6 (0.9)	13 (2.0)

*Clinically meaningful difference (≥5% absolute difference) between placebo and cladribine tablets 3.5 mg/kg

TEAE, treatment-emergent adverse event

Supplementary Table S4. TEAEs leading to treatment discontinuation occurring within the first 2, 6 and 12 weeks – Combined CLARITY and ORACLE-MS (ordered by system organ class and preferred term)

n (%)	Placebo (N=641)			Cladribine tablets 3.5 mg/kg (N=636)		
	First 2 weeks	First 6 weeks	First 12 weeks	First 2 weeks	First 6 weeks	First 12 weeks
Patients with any TEAE leading to treatment discontinuation	3 (0.5)	6 (0.9)	9 (1.4)	2 (0.3)	6 (0.9)	10 (1.6)
Blood and lymphatic system disorders	-	-	-	1 (0.2)	2 (0.3)	3 (0.5)
Lymphopenia	-	-	-	1 (0.2)	2 (0.3)	3 (0.5)
General disorders and administration site conditions	-	1 (0.2)	1 (0.2)	-	-	-
Chest pain	-	1 (0.2)	1 (0.2)	-	-	-
Hepatobiliary disorders	-	-	-	-	1 (0.2)	1 (0.2)
Hepatitis toxic	-	-	-	-	1 (0.2)	1 (0.2)
Infections and infestations	2 (0.3)	2 (0.3)	3 (0.5)	-	-	-
Appendicitis	1 (0.2)	1 (0.2)	1 (0.2)	-	-	-

Treatment-emergent adverse events occurring early in the treatment course of cladribine tablets in two Phase 3 trials in multiple sclerosis

Gardnerella infection	1 (0.2)	1 (0.2)	1 (0.2)	-	-	-
Varicella	-	-	1 (0.2)	-	-	-
Investigations	-	-	1 (0.2)	-	2 (0.3)	3 (0.5)
Alanine aminotransferase increased	-	-	1 (0.2)	-	1 (0.2)	2 (0.3)
Aspartate aminotransferase increased	-	-	1 (0.2)	-	-	1 (0.2)
Lymphocyte count abnormal	-	-	-	-	1 (0.2)	1 (0.2)
Metabolism and nutrition disorders	-	1 (0.2)	1 (0.2)	-	-	-
Anorexia	-	1 (0.2)	1 (0.2)	-	-	-
Musculoskeletal and connective tissue disorders	-	-	-	-	-	1 (0.2)
Rheumatoid arthritis	-	-	-	-	-	1 (0.2)
Respiratory, thoracic, and mediastinal disorders	-	1 (0.2)	1 (0.2)	-	-	-
Cough	-	1 (0.2)	1 (0.2)	-	-	-

Skin and subcutaneous tissue disorders	-	-	1 (0.2)	1 (0.2)	1 (0.2)	2 (0.3)
Dermatitis	-	-	-	1 (0.2)	1 (0.2)	1 (0.2)
Dermatitis allergic	-	-	-	-	-	1 (0.2)
Urticaria	-	-	1 (0.2)	-	-	-
Pregnancy, puerperium and perinatal conditions	-	1 (0.2)	1 (0.2)	-	-	-
Pregnancy	-	1 (0.2)	1 (0.2)	-	-	-
Social circumstances	1 (0.2)	1 (0.2)	1 (0.2)	-	-	-
Pregnancy of partner	1 (0.2)	1 (0.2)	1 (0.2)	-	-	-

TEAE, treatment-emergent adverse event

Supplementary Table S5. TEAEs leading to treatment discontinuation occurring during the periods of the treatment weeks (Weeks 1 and 5), after completing the first treatment week and prior to the second treatment week (Weeks 2–4), and after completing the second treatment week up to Week 12 (Weeks 6–12) – Combined CLARITY and ORACLE-MS (ordered by system organ class and preferred term)

	Placebo (N=641)			Cladribine tablets 3.5 mg/kg (N=636)		
**n (%)**	**Weeks 1 and 5 (Treatment weeks)**	**Weeks 2–4 (Weeks between treatments)**	**Weeks 6–12 (Weeks after treatment)**	**Weeks 1 and 5 (Treatment weeks)**	**Weeks 2–4 (Weeks between treatments)**	**Weeks 6–12 (Weeks after treatment)**
Patients with any TEAE leading to treatment discontinuation*	4 (0.6)	-	4 (0.6)	4 (0.6)	2 (0.3)	4 (0.6)
Blood and lymphatic system disorders*	-	-	-	2 (0.3)	-	1 (0.2)
Lymphopenia*	-	-	-	2 (0.3)	-	1 (0.2)
General disorders and administration site conditions	-	-	1 (0.2)	-	-	-
Chest pain	-	-	1 (0.2)	-	-	-
Hepatobiliary disorders	-	-	-	-	1 (0.2)	-
Hepatitis toxic	-	-	-	-	1 (0.2)	-
Infections and infestations	2 (0.3)	-	1 (0.2)	-	-	-
Appendicitis	1 (0.2)	-	-	-	-	-
Gardnerella infection	1 (0.2)	-	-	-	-	-

Varicella	-	-	1 (0.2)	-	-	-
Investigations	-	-	1 (0.2)	1 (0.2)	1 (0.2)	1 (0.2)
Alanine aminotransferase increased	-	-	1 (0.2)	-	-	1 (0.2)
Aspartate aminotransferase increased	-	-	1 (0.2)	-	-	1 (0.2)
Lymphocyte count abnormal	-	-	-	-	1 (0.2)	-
Metabolism and nutrition disorders	1 (0.2)	-	-	-	-	-
Anorexia	1 (0.2)	-	-	-	-	-
Musculoskeletal and connective tissue disorders	-	-	-	-	-	1 (0.2)
Rheumatoid arthritis	-	-	-	-	-	1 (0.2)
Respiratory, thoracic, and mediastinal disorders	1 (0.2)	-	-	-	-	-
Cough	1 (0.2)	-	-	-	-	-
Skin and subcutaneous tissue disorders	-	-	1 (0.2)	1 (0.2)	-	1 (0.2)
Dermatitis	-	-	-	1 (0.2)	-	-
Dermatitis allergic	-	-	-	-	-	1 (0.2)
Urticaria	-	-	1 (0.2)	-	-	-

Pregnancy, puerperium and perinatal conditions	-	1 (0.2)	-	-	-	-
Pregnancy	-	1 (0.2)	-	-	-	-
Social circumstances	1 (0.2)	-	-	-	-	-
Pregnancy of partner	1 (0.2)	-	-	-	-	-

*Indicates differences of ≥2 patients between placebo and cladribine tablets 3.5 mg/kg during each period
